# Management of Giant Gastric Ulcer Perforation: Report of a Case and Review of the Literature

**DOI:** 10.1155/2016/4681989

**Published:** 2016-12-06

**Authors:** Nitin Vashistha, Dinesh Singhal, Gurpreet Makkar, Suneel Chakravarty, Vivek Raj

**Affiliations:** ^1^Department of Surgical Gastroenterology, Max Super Speciality Hospital, Saket, New Delhi 110017, India; ^2^Department of Radiology, Max Super Speciality Hospital, Saket, New Delhi 110017, India; ^3^Department of Gastroenterology & Hepatology, Max Super Speciality Hospital, Saket, New Delhi 110017, India

## Abstract

Giant gastric ulcer (GGU) is defined as an ulcer more than 3 cm in diameter. Now infrequent in clinical practice, in the pre-H2 receptor antagonist (H2RA) era, the incidence of GGU varied between 12 and 24% of all gastric ulcers. Proton pump inhibitors reportedly achieve better healing rates and symptom relief in comparison to H2RA. The GGU is associated with high incidence of serious complications such as hemorrhage. A perforated GGU though rare (<2%) offers serious challenges in management. We report one such case wherein the role of multidetector CT scan (MDCT) for diagnosis and treatment planning, surgical options for GGU perforations, and factors affecting outcome are discussed.

## 1. Giant Gastric Ulcer Perforation

Giant gastric ulcer has become an infrequent entity in the modern day clinical practice following the advent of proton pump inhibitors (PPI). The associated complications include hemorrhage, malignancy, and general ill health. Perforation of GGU though rare presents unique challenges in management especially if the patient has serious comorbidity or presents late.

A 58-year-old gentleman presented to the emergency room with 5-day history of abdomen pain, distension, and altered sensorium and 1-day history of decreased urine output. There was a history of heavy alcohol intake, chain smoking, and consumption of large doses of NSAIDS till 1 day prior to admission.

At general physical examination, his vitals were stable but he was disoriented. The abdomen was distended with rebound tenderness, tympanic note, and absent bowel sounds.

**Figure 1:**
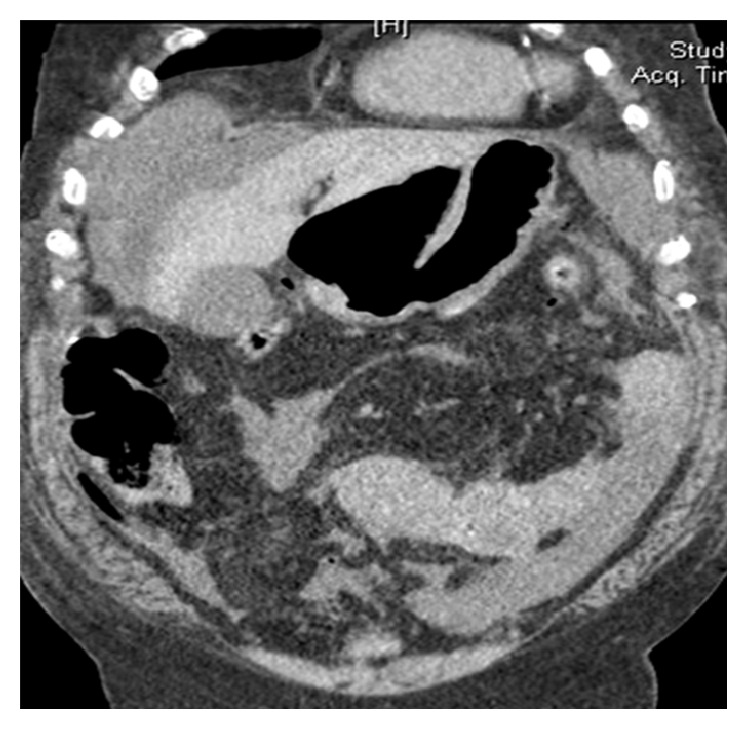
Computed tomography scan of abdomen showing discontinuity of the anterior wall of the gastric antrum with pneumoperitoneum.

With a clinical diagnosis of peritonitis, the patient was investigated. An emergency noncontrast CT scan of the abdomen performed on 64-slice multidetector MDCT scan revealed a large (>3 cm) discontinuity of the anterior wall of the gastric antrum with pneumoperitoneum suggestive of giant gastric perforation (Figure 1). The other significant positive blood investigations included white cell count of 11600/mm^3^, deranged kidney function tests (serum creatinine 2.7 mg/dL and pH 7.287), and serum procalcitonin of 23.59 ng/mL.

**Figure 2:**
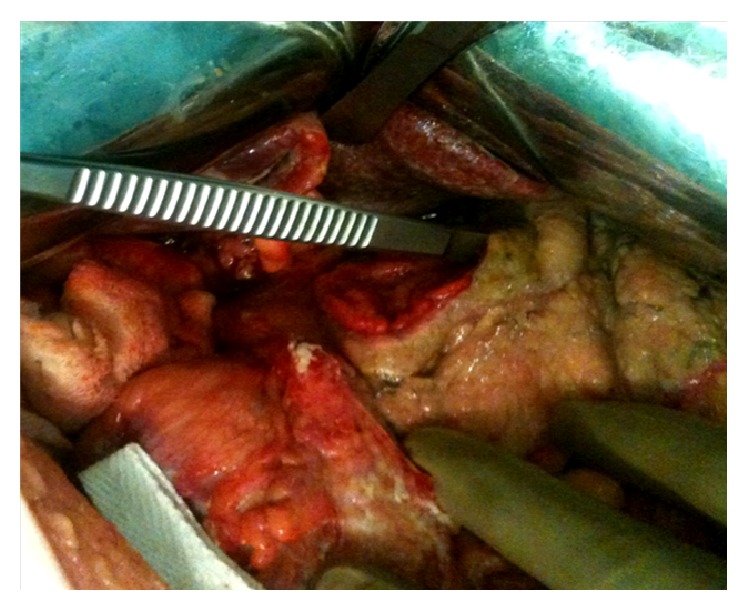
Operative picture showing giant gastric perforation.

Following adequate resuscitation, the patient was taken up for emergency laparotomy. Operative findings confirmed a 5 × 5 cm perforation of the gastric antrum (Figure 2). A distal gastrectomy with Billroth-II reconstruction with feeding jejunostomy was performed. The subsequent histopathological examination did not reveal any malignancy.

In the postoperative period, the patient remained sick and required regular hemodialysis and high inotropes and ventilator support. Despite the best available multidisciplinary care, the patient died on the 7th postoperative day.

## 2. Discussion

Our case report highlights several important issues in the management of perforated GGU including limited surgical options, the current role of MDCT for diagnosis and treatment planning, and factors affecting outcome.

Giant gastric ulcer (GGU) has been defined as an ulcer >3 cm in diameter or large enough to occupy at least one wall [1, 2]. The incidence of GGU in the pre-H2 receptor antagonist (H2RA) era varied between 12 and 24% of all gastric ulcers [2, 3]. A subsequent meta-analysis that analyzed comparative efficacy of H2RA versus PPI reported that the latter achieves better healing rates and greater relief of symptoms for gastric ulcers [4]. The clinical importance of this entity was due to its intractability (necessitating surgery) and higher incidence of serious complications such as hemorrhage (12–44%) and malignancy (10–20%) [2, 3]. The associated long-term mortality, often due to unrelated causes, was high, indicating that these patients were more seriously ill as compared to the ones with smaller ulcers [2]. A perforated GGU is infrequent with a reported incidence of 02/129 (1.5%) [2].

Giant gastric ulcer perforations present formidable challenges in management. This is particularly true when the patient is elderly or presenting late (more than 24 hours) or with multiorgan failure. Over the years, partial gastrectomy (PG) and omental plugging (OP) have emerged as preferred surgical options. Experience with other procedures such as serosal patch, free jejunal pedicle flap, and partition gastrectomy is limited.

The important issues that merit consideration while managing perforated GGU include exclusion of malignancy and reducing recurrence of ulcer.

Partial gastrectomy is the only procedure that achieves both objectives. It demands more technical expertise and requires longer operating times and blood transfusions. The PG is reported to provide lower recurrence rates in the long term though the perioperative mortality was higher [5].

The OP with placement of drains and feeding jejunostomy is a safe and reliable procedure [6]. It has the advantage of technical simplicity and can be performed expeditiously. Hence, OP may be the preferred option in critically ill patients especially where technical expertise/facilities are limited.

In difficult situations wherein omental plugging is not deemed feasible and technical expertise for PG is limited, jejunal serosal patch may be considered as an alternative.

Laparoscopy is being increasingly employed in the management of GGU perforations. Laparoscopic OP may be a technically simple procedure. Laparoscopic PG in this setting has been shown to be technically feasible with acceptable outcomes but this operation should be restricted to experts with advanced laparoscopic skills.

Over the last decade, MDCT has emerged as a valuable modality for the diagnosis and determination of site GI of tract perforation [7]. Besides the presence of pneumoperitoneum, the other significant CT scan findings that help in localizing the site of perforation include concentration of extraluminal air bubbles in close proximity to the perforated viscus, focal bowel wall thickening, and focal discontinuity of the bowel wall. Extravasation of oral contrast on CT scan is considered diagnostic of intestinal perforation. However, the reported sensitivity is low and ranges between 19 and 42%. Hence, the use of oral contrast may not give much additional information and may delay the surgery [8].

In our patient, additional information pertaining to the size of perforation (>3 cm) was also made available. This resulted in the preoperative diagnosis of GGU perforation. The surgical team planned for a distal gastrectomy and appropriately counseled the family also.

Despite the advances in critical care, anesthesia, and surgical techniques, the 30-day postprocedure mortality of perforated peptic ulcer ranged between 16 and 26% at the best of the centers [9, 10]. Some of the predictors of adverse outcome include age more than 60 years, shock at the time of admission, multiorgan failure, and delay in presentation and in performing surgery [10].

In summary, GGU perforation is a rare entity in current day clinical practice but is one that is associated with high morbidity and mortality. A noncontrast MDCT may aid preoperative diagnosis and treatment planning. The choice of operative procedure (PG or OP) is determined by patient presentation and available local technical skills and facilities.
